# Postbiotic Fractions of Probiotics *Lactobacillus plantarum* 299v and *Lactobacillus rhamnosus* GG Show Immune-Modulating Effects

**DOI:** 10.3390/cells12212538

**Published:** 2023-10-28

**Authors:** Agnieszka Magryś, Mateusz Pawlik

**Affiliations:** Chair and Department of Medical Microbiology, Medical University of Lublin, ul. Chodźki 1, 20-093 Lublin, Poland

**Keywords:** probiotics, gut inflammation, co-culture, *Lactobacillus plantarum* 299v, *Lactobacillus rhamnosus* GG, postbiotics

## Abstract

Probiotic bacteria belonging to *Lactobacillus* spp. are important producers of bioactive molecules, known as postbiotics, that play essential roles in the immunological support of the intestinal mucosa. In this study, the system of co-culture of intestinal epithelial cells with macrophage cells in vitro was used to study the potential effect of postbiotic fractions of *L. rhamonosus* and *L. plantarum* on the modulation of the immune response induced by pro-inflammatory stimuli. This study’s results revealed that the presence of probiotic bacterial components on the mucosal surface in the early and late stage of inflammatory conditions is based on cellular interactions that control inflammation and consequent damage to the intestinal epithelium. In our studies, heat killed fractions of probiotic bacteria and their extracted proteins showed a beneficial effect on controlling inflammation, regardless of the strain tested, consequently protecting intestinal barrier damage. In conclusion, the presented results emphasize that the fractions of probiotic bacteria of *L. plantarum* and *L. rhamnosus* may play a significant role in the regulation of LPS-mediated cytotoxic activity in intestinal epithelial cells. The fractions of probiotic strains of *L. rhamnosus* and *L. plantarum* showed the potential to suppress inflammation, effectively activating the anti-inflammatory cytokine IL-10 and modulating the IL-18-related response.

## 1. Introduction

The intestinal microbiome bears a significant role in the function and integrity of the gastrointestinal tract. It contributes to the inhibition and elimination of potential pathogens, maintains a symbiotic relationship with the gut mucosa and confers protective functions for GI physiological homeostasis [1]. Intestinal microbiota, which function in symbiotic or pathogenic relationships with a host, can also influence the local immune system to stimulate immune cells, causing tissue damage and ultimately leading to chronic inflammation. For this reason, a disturbed imbalance between the functional composition and metabolic activities of the microbial community, known as dysbiosis, is a contributory cause for a range of metabolic and inflammatory diseases in humans, including cancer and colitis [2].

Analyzing the importance of dysbiosis-driven diseases, it is accepted that probiotics can be used to introduce beneficial effect on inflammatory responses in the GI tract or support the functionality of resident gut microbiota [3,4,5]. Indeed, the use of probiotic bacteria in a preventive or therapeutic strategy may restore the balance of the intestinal environment and mitigate the pathogenesis of inflammatory diseases by modifying immune responses [6]. Some of the beneficial properties of probiotics are due to the bioactive substances they secrete, the end products of metabolism (such as short-chain fatty acids, enzymes, proteins, organic acids, vitamins and amino acids), or the cell wall components [7,8,9]. These bioproducts, known as postbiotics, have been found to have similar functions to probiotics and their health-promoting effects may be similar [8,9]. They stimulate the intestinal microbiome and support intestinal immune functions by controlling the balance between two major arms of the immune system, Th1 and Th2 lymphocytes, thus minimizing intestinal inflammation and restoring homeostasis to the gut environment [9]. However, the exact molecular interactions between postbiotics and the host through which they express their positive effects have not been fully elucidated [2,8,9,10].

The immunomodulation by postbiotics is partly related to the strain of probiotic used [8]. In particular, lactic acid bacteria, including *Lactobacillus* spp. are important producers of a wide range of highly effective bioproducts [9].

Although the beneficial properties of postbiotics derived from *Lactobacillus* species are well known, the mechanisms underlying their interaction with immune cells, influencing immunomodulation, still need to be better understood [7,8]. Balanced communication between intestinal epithelial cells, immune cells and the intestinal microbiome is essential to maintain intestinal homeostasis [11]. The complexity of the mucosal immune system, however, is difficult to mimic in vitro, but with the help of a co-culture system, the mechanisms involved in communication between epithelial cells and immune cells can be investigated. Therefore, in this study, the system of co-culture of intestinal epithelial cells (Caco-2) with macrophage cells (activated THP-1) in vitro was used to study the potential effect of postbiotics secreted by *L. plantarum* and *L. rhamnosus* on the modulation of the immune response induced by pro-inflammatory stimuli.

## 2. Materials and Methods

### 2.1. Bacterial Strains and Culture Conditions

*Lactobacillus rhamnosus* GG (ATCC 53103) and *Lactobacillus plantarum* 299v were obtained from the commercially available dietary supplement Sanprobi IBS (*L. plantarum*) and Dicoflor 6 (*L. rhamnosus*). Probiotic bacterial strains were cultured under aerobic conditions on deMan Rogosa Sharp (MRS) medium (Oxoid Ltd., Basingstoke, UK) at 37 °C for 24 h.

### 2.2. Preparation of Thermally Killed Bacterial Cells

Heat-killed (HK) bacterial cells were prepared according to a modified method described by Young et al. [12]. Briefly, after the bacterial cells reached the stationary phase in MRS broth (10–14 h at 37 °C with shaking at 120 rpm), they were centrifuged at 5000× *g* for 10 min, washed with phosphate-buffered saline (PBS, pH 7) and adjusted to 1 × 10^10^ CFU/mL. Next, the cells were heated for 2 h at 90 °C. Bacterial cell death was confirmed by plating the suspensions onto MRS agar and incubating for a minimum of 18 h. The integrity of the bacterial cells was analyzed by Gram staining.

### 2.3. Preparation of Protein Extracts Secreted by Lactobacillus spp.

A single colony of fresh culture of each probiotic strain was used to inoculate 10 mL of MRS broth and incubated overnight at 37 °C with shaking. The culture (0.5 mL) was then used to inoculate 50 mL of fresh MRS medium and incubated overnight, until a stationary phase was reached. The bacterial culture supernatants or fresh MRS broth (control) were then collected by centrifugation at 3500× *g* for 10 min and filtered through 0.45 μm nitrocellulose filters. An amount of 10 mL of 5% *v*/*v* sodium deoxycholate was added to the resulting filtrate to remove salts, and then incubated at 4 °C for 30 min. Proteins were precipitated by adding chilled trichloroacetic acid (TCA) (final concentration of 60 g/L) and held for 2 h at 4 °C. The precipitates were recovered by centrifugation (9300× *g* for 10 min, 4 °C). The protein pellets were washed twice with 2 mL of chilled acetone and allowed to dry at room temperature. The proteins were re-dissolved in an ultrasonic bath (35 kHz frequency) for 2 min in 1 mL of sterile PBS. Bradford’s method was used to estimate the protein concentration in each extract secreted by the bacteria.

### 2.4. Evaluation of the Role of Probiotic Bacteria in the Modulation of the Immune Response by Macrophages

The immunomodulatory effect of probiotic fractions (secreted proteins SP and heat-killed bacteria HK) on immune cells was tested using macrophages derived from THP-1 cells (ATCC TIB-202). For this purpose, macrophages (1 × 10^5^ cells/100 μL/well) were pre-treated with *L. plantarum* and *L. rhamnosus* fractions: 3 × 10^8^ CFU/mL of HK or 3 μg/mL SP, for 18 h and then stimulated with 100 ng/mL LPS and incubated for 18 h at 37 °C, 5% CO_2_. The macrophage cell supernatant was then harvested and stored at −20 °C until cytokine detection by ELISA.

### 2.5. Co-Culture of Epithelial Cells and Macrophages

This study used a co-culture model of epithelial cells (Caco-2, ATCC HTB-37) and macrophages (activated THP-1, ATCC TIB-202) to mimic intestinal inflammation induced by inflammatory signal (LPS) in the presence of postbiotics. Two groups of Caco-2 cells were plated on culture inserts (0.4 μm pore size) at a cell density of 5 × 10^5^ cells/500 μL/well and cultured in DMEM in a 5% CO_2_ incubator at 37 °C for 21 days allowing full cell differentiation. One group of Caco-2 cells cultured on inserts was incubated with macrophages for 18 h in a co-culture system, where macrophages were placed in the lower chamber of the culture plate, facing the basolateral side of the epithelial monolayer. The second group of Caco-2 cells were left as controls.

Epithelial cells were stimulated apically with HK or SP fractions for 18 h. To mimic the inflammatory bowel pathology, epithelial cells in a co-culture system were stimulated basolaterally with 100 ng/mL LPS for 4 h or 18 h in the presence or absence of HK or SP fractions. The apical cell supernatant was collected and stored at −20 °C and used further to detect cytokine synthesis by ELISA.

### 2.6. ELISA for Cytokines

The level of IL-18 and IL-10 in the supernatant of cell cultures (macrophages, Caco-2, and Caco-2/THP-1 co-culture) stimulated with the tested bacterial fractions was determined using commercially available ELISA detection kits (Human IL-18 and IL-10 ELISA kit, Diaclone), following the manufacturer’s instructions.

### 2.7. Determination of Cytotoxicity Using the Lactate Dehydrogenase [LDH] Release Assay

To confirm that the tested bacterial metabolites were not toxic for cultured immune cells, cell viability was assessed using commercially available Pierce LDH Cytotoxicity Assay Kit (Thermo Scientific, Waltham, MA, USA), following the manufacturer’s instructions. The specific cytotoxicity was calculated using the following formula:

% cytotoxicity = (tested LDH activity − control LDH activity)/(maximum LDH activity − spontaneous LDH activity) × 100

Relative amounts of LDH release were measured (absorbance at 490 nm) using an ELISA plate reader. The tests were carried out in 3 independent replicates.

### 2.8. Statistical Analysis

Statistical analyses of the obtained results were performed using a two-tailed unpaired *t*-test (2 groups) or ANOVA with Tukey’s post hoc test (multiple groups). *p* < 0.05 was considered statistically significant. All results are expressed as means ± SD. All experiments were performed in 3 independent replicates (*n* = 3).

## 3. Results

### 3.1. Anti-Cytotoxic Activity of Heat-Killed Probiotic Bacteria and Their Protein Extracts in Monoculture Models

**Figure 1:**
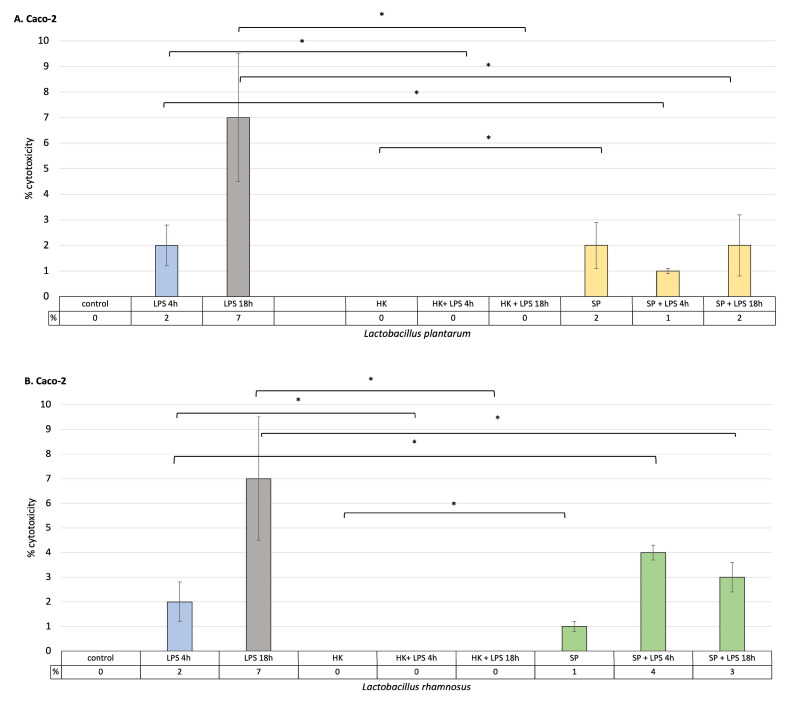

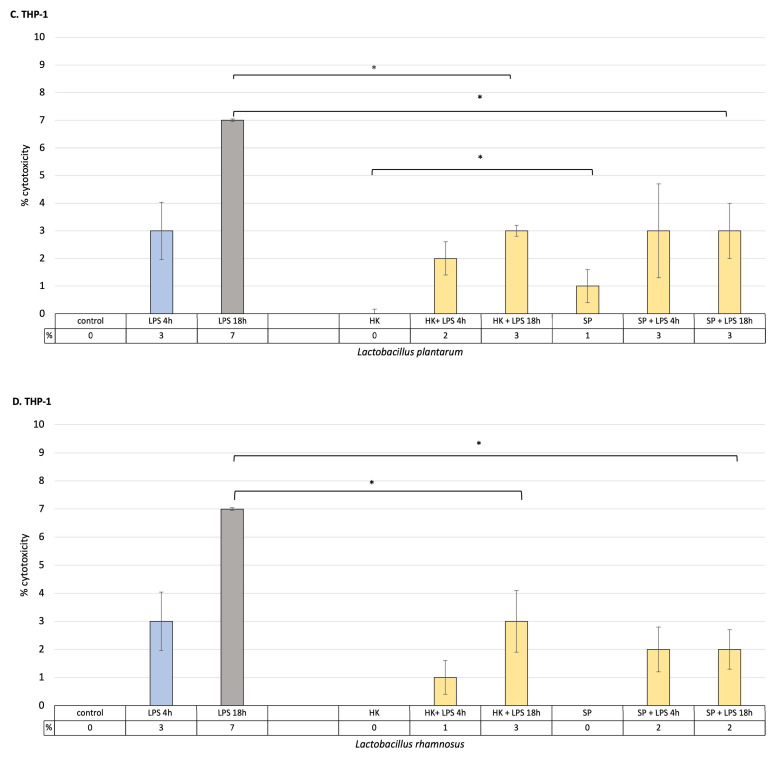
The effect of heat-killed probiotic bacteria and their protein extracts on the degree of LDH release from cells stimulated with LPS for 4 h or 18 h. (**A**) % cytotoxicity, as measured by the amount of LDH released into the cell culture medium, relative to the Caco-2 cell line in the presence or absence of the *L. plantarum* fractions; (**B**) % cytotoxicity, as measured by the amount of LDH released into the cell culture medium, relative to the Caco-2 cell line in the presence or absence of the *L. rhamnosus* fractions (**C**)% cytotoxicity, as measured by the amount of LDH released into the cell culture medium, relative to the activated THP-1 cell line (macrophages) in the presence or absence of the *L. plantarum* fractions; (**D**) % cytotoxicity, as measured by the amount of LDH released into the cell culture medium, relative to the activated THP-1 cell line line in the presence or absence of the *L. rhamnosus* fractions. Graphs represent mean ± SD values obtained from 3 independent experiments (*n* = 3); * indicates significant statistical difference at *p* < 0.05.

As a result of LPS stimulation of intestinal epithelial cells and macrophages, it was observed that the percentage of cell cytotoxicity increased with time. At the same time, heat-killed *L. plantarum* and *L. rhamnosus* did not induce the release of LDH into the cell culture medium. In addition, killed cells of both species effectively reduced cytotoxicity after 4 h and 18 h of stimulation with LPS, and the level of LDH was statistically significantly lower compared to control cells treated with LPS alone in both macrophage and Caco-2 cell cultures (*p* < 0.05). The secreted protein metabolites of *L. rhamnosus* and *L. plantarum* had anti-cytotoxic effects on intestinal epithelial cells and macrophages previously treated with LPS for 18 h (Figure 1). Both bacterial strains showed a significant level of LDH reduction in intestinal epithelial cells and macrophages cultures stimulated with LPS (*p* < 0.05). This proves the effectiveness of these strains in preventing cell damage caused by the induction of an immune response. *Lactobacillus plantarum* showed greater anti-cytotoxic effectiveness than *L. rhamnosus* in the initial stage of inflammation, after 4 h of LPS treatment. Here, it was shown that the level of LDH released by intestinal epithelial cells is lower in the case of *L. plantarum*, while in cultured macrophages, it is lower in the presence of *L. rhamnosus*. Both of the strains used significantly reduced the levels of LDH released compared to the control. These results may suggest that the greatest effectiveness in reducing inflammation, both in the initial phase of inflammation (4 h) and in the late phase (18 h), can be obtained by using both bacterial strains simultaneously.

### 3.2. The Role Lactobacillus Fractions in LPS-Induced Modulation of IL-18 Secretion in Monoculture of Caco-2 and Activated THP-1 Cell Lines and in the Caco-2/Macrophage Co-Culture Model

**Figure 2:**
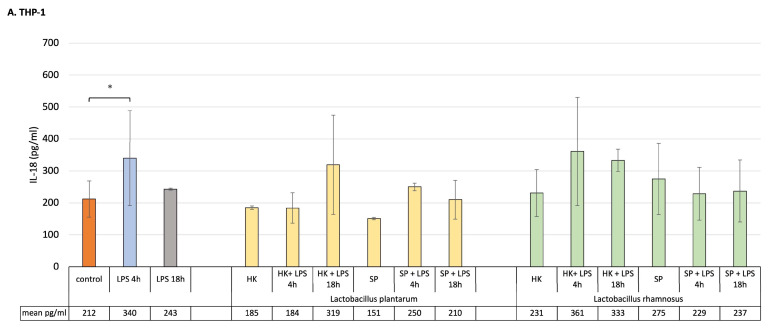

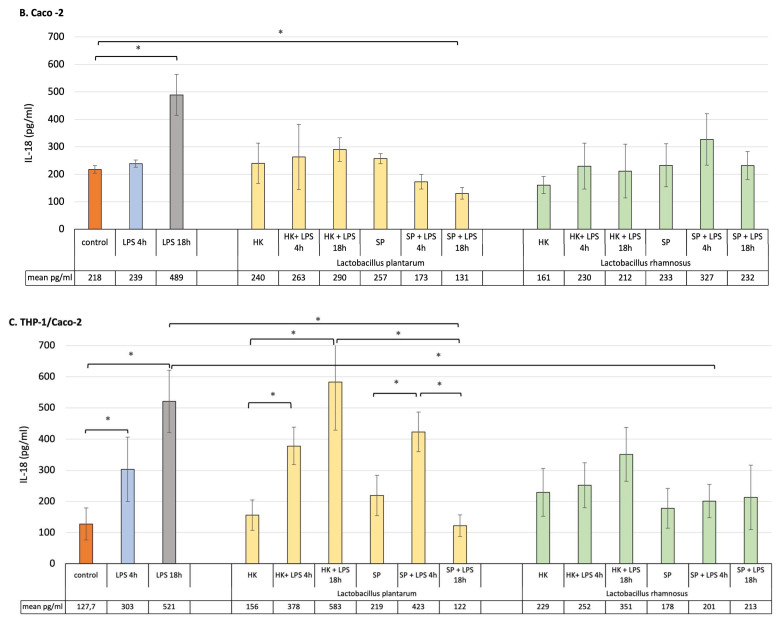
Production of IL-18 by (**A**) macrophages, (**B**) Caco-2 cells and (**C**) Caco-2/macrophage co-cultures stimulated by fractions of probiotic bacteria in response to LPS induction (4 h and 18 h). Graphs represent mean ± SD values obtained from 3 independent experiments (*n* = 3); * means significant statistical difference at *p* < 0.05.

For this study, to mimic the early and late inflammatory state, a THP-1 macrophage cell line and Caco-2 cells pre-treated with lactic bacteria fractions were treated with LPS for 4 h, in order to reflect early inflammation, and for 18 h, simulating late inflammation conditions. As shown in Figure 2A, short incubation of macrophages with LPS significantly increased the concentration of IL-18, but 18 h incubation had no significant impact on the level of the cytokine release. The results demonstrated that the HK and SP of *L. plantarum* and *L. rhamnosus* alone did not significantly affect IL-18 concentration. Also, the level of the cytokine did not significantly change from pre-treatment of THP-1 macrophages with HK or SP of both probiotic bacterial strains, remining at the level observed for control cells.

Intestinal epithelial cells in monoculture in most cases did not show significant differences in IL-18 production after being stimulated with products of probiotic bacteria or LPS. However, after their treatment with LPS for 18 h, the level of IL-18 significantly increased compared to control unstimulated culture. Finally, only SP of *L. plantarum* were able to significantly calm the inflammation state by decreasing the level of IL-18 in the monoculture conditions (Figure 2B).

Compared to both macrophages and Caco-2 cell monocultures, a clear increase in IL-18 production was detected in the co-culture model as a response to LPS induction (Figure 2C). The results indicated that, in the presence of SP and HK of the probiotic strain of *L. plantarum*, the level of pro-inflammatory cytokine significantly increased during the acute inflammation, whereas the induced chronic state of inflammation was suppressed by SP but not by HK of *L. plantarum. Lactobacillus rhamnosus* pre-treatment had no impact on the production of IL-18 in the co-culture model, with the exception of the SP of the bacterium that significantly decreased IL-18 concentration after LPS induction for 18 h.

### 3.3. The Role of SP and HK of L. plantarum and L. rhamnosus in LPS-Induced Modulation of Anti-Inflammatory IL-10 Secretion in Monoculture of Caco-2 and Activated THP-1 Cell Lines and in the Caco-2/Macrophage Co-Culture Model

To estimate the strains’ capacity to inhibit an LPS-induced inflammatory state, the level of anti-inflammatory IL-10 cytokine was measured in monoculture of macrophages and intestinal epithelial cells as well as in the co-culture models.

**Figure 3:**
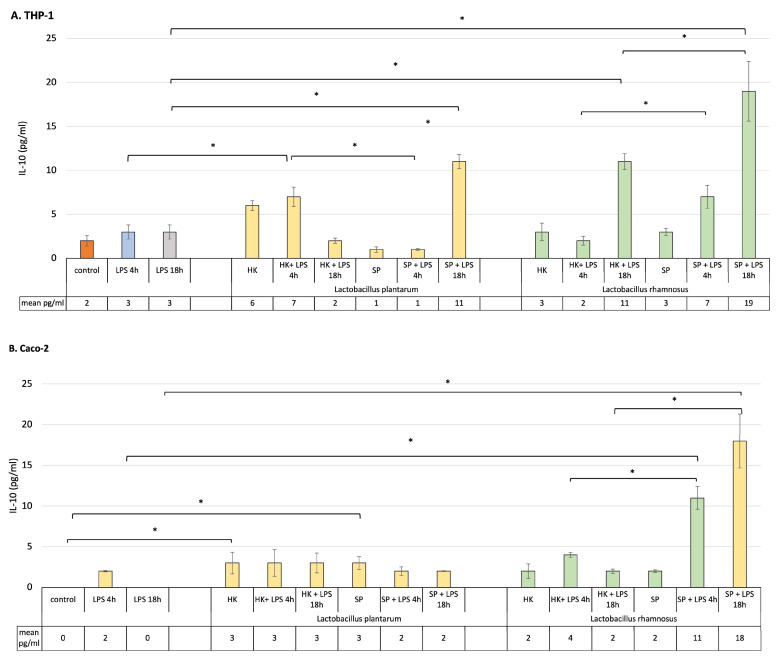

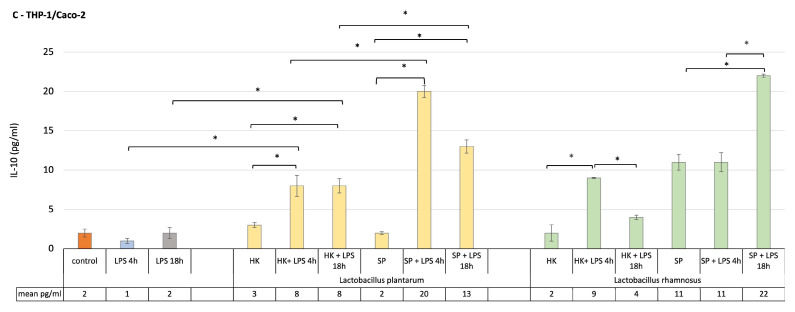
Production of IL-10 by (**A**) macrophages, (**B**) Caco-2 cells and (**C**) Caco-2/macrophage co-cultures stimulated by lactic bacteria fractions in response to LPS induction (4 h and 18 h). Graphs represent mean ± SD values obtained from 3 independent experiments (*n* = 3); * indicates significant statistical difference at *p* < 0.05.

The presence of probiotic bacteria fractions before introduction of the inflammatory signal to the culture did not influence significantly the level of IL-10 in THP-1-activated macrophages or Caco-2 cells (Figure 3A,B). After brief exposure of macrophages to LPS, the HK of both probiotic bacterial strains significantly increased the production of IL-10, when compared to the cells stimulated with LPS for 4 h only. At the same time, SP of the lactic bacteria have no significant influence on the level of the anti-inflammatory cytokine. When an inflammatory state was induced in macrophages for 18 h, the SP of *L. plantarum* and *L. rhamnosus* induced a significant elevation of the IL-10 by the sensitized cells. Also, the HK cells of *L. rhamnosus* significantly influenced the production of IL-10 when macrophages were in contact with LPS for 18 h. Interestingly, SP of *L. rhamnosus* but not *L. plantarum* were able to create an anti-inflammatory environment in a monoculture of Caco-2 cells independently, after 4 h or 18 h of LPS stimulation (Figure 3B).

In order to find out the role of postbiotics’ pre-treatment in the modulation of the anti-inflammatory, protective environment, a co-culture model was used to perform this task. Data presented in Figure 3C show the effect of the treatment on modulation of IL-10 production in Caco-2/THP-1 macrophage co-culture. In the normal, homeostatic co-culture model, all probiotic fractions non-significantly upregulated the production of anti-inflammatory IL-10. In the presence of inflammatory stimuli for 4 h, the level of IL-10 was significantly augmented when the cells were pre-treated with the fractions of probiotic bacteria in all cases. The levels were higher when compared to the control unstimulated and postbiotics-only-treated cells. Additionally, the SP of both bacterial strains maintained the anti-inflammatory condition at a higher level than HK.

In response to LPS stimulation for 18 h, prior pre-treatment with probiotic bacteria products affected IL-10 production in various ways. Even though the level of this cytokine was elevated, compared to the culture in which the cells were only subjected to LPS induction for 18 h, it was lower than when the cells were treated with LPS for 4 h. The only exception was *L. rhamnosus*, whose SP raised the level of IL-10 to the highest statistically level compared to other bacterial products.

## 4. Discussion

Growing evidence indicates that the interaction between probiotics and intestinal epithelial cells modulates many aspects of the innate and adaptive immune responses and deserves special attention for its impact on consumer’s health. Many recent studies focus on the potential of probiotics for health promotion, disease prevention and use as a treatment strategy for various immune-mediated diseases. The beneficial immunomodulatory effects of probiotics, along with their disease-fighting properties, are highly strain-specific and vary from host to host as they are influenced by age, sex, and disease state. Live probiotic bacteria, but also their metabolites or soluble mediators, affect the strengthening of the integrity of the intestinal epithelial barrier and antigen-presenting cells, including dendritic cells and macrophages, both directly and indirectly [13,14,15]. Depending on the type of probiotic strain and the cell it affects, these immunomodulatory effects may manifest as activation or suppression of the immune system [16].

In this study, two probiotic bacterial strains were used, *Lactobacillus rhamnosus* GG (ATCC 53103) and *Lactobacillus plantarum* 299v, as they have been previously reported to prevent gastrointestinal infections by attaching to the surface of the intestinal mucosal barrier, creating less opportunities for pathogenic microorganisms to grow [14,17]. They are also believed to alleviate intestinal damage and inflammation by inhibiting epithelial cell apoptosis, increasing mucin production and modulate the immune response of intestinal lymphoid and epithelial cells through bacterial products and cell wall components [17]. Inflammation of the intestines causes diarrhea and other forms of intestinal disorders. The local inflammatory response can eliminate the invading pathogen and consequently heal the tissue damage. However, excessive inflammatory reactions are closely related to the development of chronic diseases. Therefore, alleviating inflammation is a key preventive and therapeutic strategy in disease control [18,19].

The aim of the study was to determine the immunomodulatory capacity of probiotic lactic acid bacteria postbiotics in LPS-induced cell culture by comparing two stages: early and late inflammation. In this study, two cell line types were used: THP-1 derived macrophages to represent immune cells and Caco-2 epithelial cell line that, after 21 days of cultivation formed a microvillous shape, resembling the morphology and function of the small intestinal barrier [20].

To clarify whether pre-treatment of cells with *Lactobacillus* strains fractions protects the GIT from LPS-induced injury and is associated with the inhibition of cytotoxicity, the level of LDH was determined in the cell culture medium of intestinal epithelial cells and macrophages treated with LPS for 4 h and 18 h. LDH is a general indicator of acute or chronic tissue damage and is considered a marker of inflammation [21]. It is an enzyme permanently present in the cytoplasm and released from cells with a damaged membrane. Therefore, LDH activity in the cell culture medium is positively correlated with the number of necrotic cells. It was demonstrated that LPS infection alone, but not postbiotic treatment, increased cytotoxicity in intestinal cells and macrophages. In general, pre-treatment of cells with probiotic fractions had a positive effect on their survival after induction with an inflammatory factor for 4 h as well as 18 h. Both SP and HK of both strains of probiotic bacteria significantly reduced the level of cytotoxicity to macrophages and intestinal epithelial cells. The results suggest that prophylaxis with lactic bacteria bioproducts can effectively attenuate the increased cytotoxicity that accompanies both early and late LPS-induced intestinal epithelial cell damage. Analyzing the results obtained by other authors, it seems clear that probiotics protect the intestinal cells from death in various ways. Chaoqun Han et al. provided evidence that LPS could induce the upregulation of autophagy activity, while treatment with probiotics decreased autophagy and alleviated intestinal epithelial cell injury [22]. The group of Yichao Hou analyzed the most frequently modulated genes and the pathways related to oxidative stress that are regulated by the *L. plantarum* J26 and *L. rhamnosus* GG strains. They found that in the apoptosis signaling pathway, five genes were downregulated by *L. rhamnosus* and 3 by *L. plantarum* J26 [23]. Similarly, Fang Yan reported that *L. rhamnosus* GG prevents cytokine-induced apoptosis. Moreover, products recovered from the probiotic culture broth supernatant showed concentration-dependent activation of anti-apoptotic Akt/protein kinase B and inhibition of cytokine-induced apoptosis [24].

To understand the beneficial effects of probiotic bacteria on host gastrointestinal cells, it is important to study epithelial cells and lamina propria immune cells separately, as they may play different roles in defense and immunomodulation [23]. However, as the gut is a complex system, in order to better mimic the in vivo condition of the intestine under controlled inflammation and to elucidate the immunoregulatory role of the probiotic bacterial fractions, a co-culture of Caco-2 and THP-1 macrophages was established, where intestinal epithelial cells coexist and interact with immune cells, such as macrophages. The ability of postbiotics derived from *L. plantarum* and *L. rhamnosus* strains to control short- and long-term inflammation in the gastrointestinal tract, and their effects on intestinal epithelial cells and immune cells were assessed by measuring the release of pro- and anti-inflammatory cytokines in two culture systems: monoculture and co-culture of Caco-2 cells and activated macrophages. Cytokines are mediators that primarily control and regulate immune response and inflammatory reactions but also influence epithelial cell functions. One of the most important anti-inflammatory cytokines in balancing intestinal homeostasis is IL-10. It is also recognized as a potential functional biomarker for screening the anti-inflammatory properties of probiotic cultures [13,25]. It is interesting to note that both probiotic bacterial strains stimulated anti-inflammatory cytokine production by macrophages after 4 h and 18 h of stimulation with LPS, irrelevant of the format used, heat killed or secreted proteins. In addition, *L. rhamnosus* SP was responsible for enhancing the anti-inflammatory state in epithelial cell culture, stimulating the production of large amounts of IL-10 both 4 h and 18 h after induction of Caco-2 cells with the inflammatory agent.

Several studies have demonstrated that IL-18 play a critical role in governing host–microorganism homeostasis in the intestine and is able to induce severe and chronic inflammation through induction of inflammatory mediators, such as TNFα [26,27]. The cytokine is constitutively produced by the epithelial cells of the intestine and its equilibrium is important for epithelial integrity. Otherwise, the overexpression of IL-18 is responsible for the increased susceptibility to intestinal damage. This observation correlates with clinical findings showing that an increase in the production of the cytokine correlates with the severity of irritable bowel disease [26].

In the presented study, pre-treatment of cells in monoculture with probiotic fractions did not influence the production of IL-18 after LPS stimulation, with the exception of *L. plantarum* SP decreasing its concentration in a significant manner.

Overall, the evidence indicates that the main players in the immune response during the early and late inflammatory states are macrophages which modulate the immune response by producing cytokines. Indeed, they are critical in inflammation initiation, maintenance and resolution [28]. However, epithelial cells are also an active part of mucosal innate immunity, able to produce, and more importantly, respond to cytokines [29]. This work showed that in monoculture of Caco-2 and activated THP-1 cells, HK and SP probiotic bacteria elevated the anti-inflammatory IL-10 level after LPS challenge imitating inflammation. By definition, probiotics are viable microorganisms. But, in recent years, it has been scientifically proven that certain components of probiotic bacteria, known as “postbiotics”, can also have a beneficial effect on human health. Bacterial cell wall, bacterial DNA, and bacterial bioactive components have potent immunostimulatory effects, directly interacting with host mucosal cells, leading to the modulation of signal transduction pathways [8,9,10,25,30,31]. Indeed, postbiotics can influence signal transduction processes and thus control the response of host cells to the inflammatory signal and influence immunomodulation. Yubin Li et al. proved that soluble protein HM0539, derived from *L. rhamnosus* GG, significantly inhibited the production of inflammatory factors by LPS-stimulated macrophages. The authors proved that the bioproduct they tested works by suppressing the TLR4/Myd88/NF-kB axis signaling pathway, the well-known function of which is the regulation of the inflammatory response, and its activation is a hallmark of chronic inflammation [32]. Postbiotics derived from *Lactobacillus* species have mainly immunoregulatory effects on immune-competent cells, such as macrophages. It has been found that lipoteichoic acid from the cell wall of *L. plantarum* attenuates pro-inflammatory TNF-α cytokine activation in response to LPS stimuli, but significantly upregulates IL-10 production [33]. Shi et al. also examined the effect of probiotic bacteria of the *Lactobacillus* genus on the expression of the main pro-inflammatory cytokines, TNF-α, IL-6 and L-1β, in LPS-induced macrophage cultures. They found that probiotics significantly reduced the expression of the tested pro-inflammatory cytokines in cell culture, which may be related to the inhibition of the activation of the NF-κB and MAPK signaling pathway via TLR4 [19]. In the presented study, under the influence of both HK and SP of both probiotic strains, in the environment of early and late inflammation, especially in macrophage culture, significant amounts of anti-inflammatory IL-10 were produced, while the amount of IL-18 was not changed.

In pathological conditions in the intestine, both the immune cells and epithelial cells play an important role in acute and chronic disease development. For this reason, in the next step, the role of probiotic bacterial strains in silencing and immunomodulating the inflammation in a co-culture model were evaluated. Both lactobacilli strains and all their fractions used significantly upregulated IL-10 production as a response to short and prolonged stimulation with LPS. Interestingly, only in the presence of the SP of *L. plantarum* and *L. rhamnosus* was a significant decrease in pro-inflammatory IL-18 noticed. These SP could directly interact with the host mucosal cells, leading to the modulation of signal transduction pathways and thereby protecting the intestine from an inflammatory insult, ensuring gut homeostasis and health.

This study confirms that the presence of probiotic bacterial components on the epithelial cell surface under inflammatory conditions is based on cellular interactions that control inflammation and consequent damage to the intestinal epithelium. In our studies, fractions of probiotic lactic bacteria showed a beneficial effect on controlling inflammation, regardless of the strain, consequently protecting intestinal barrier damage.

## 5. Conclusions

In conclusion, the presented results emphasize that the fractions of probiotic bacteria *L. plantarum* and *L. rhamnosus*, HK and SP, may play a role in the regulation of LPS-mediated cytotoxic activity in intestinal epithelial cells, and thus contribute to the maintenance of intestinal homeostasis. The postbiotics derived from probiotic strains of *L. rhamnosus* and *L. plantarum* showed the potential to suppress inflammation, effectively activating the anti-inflammatory cytokine IL-10 and modulating the IL-18-related response.

## Data Availability

Data sharing is not applicable to this article.
